# Acoustic Emission and Numerical Analysis of Pine Beams Retrofitted with FRP and Poplar Wood

**DOI:** 10.3390/ma13020435

**Published:** 2020-01-16

**Authors:** Francisco J. Rescalvo, María Rodríguez, Rafael Bravo, Chihab Abarkane, Antolino Gallego

**Affiliations:** 1 Building Engineering School, University of Granada, Campus Fuentenueva s/n, 18071 Granada, Spain; rescalvo@ugr.es (F.J.R.); antolino@ugr.es (A.G.); 2 Department of Structural Mechanics, University of Granada, Campus Fuentenueva s/n, 18071 Granada Spain; mariarquezm@correo.ugr.es (M.R.); rbravo@ugr.es (R.B.)

**Keywords:** acoustic emission, wood, poplar, composites, FRP

## Abstract

Acoustic emission (AE) released by pine beams retrofitted with fiber reinforced plastic (FRP) and poplar planks in bending is analyzed. Basalt fabric (FB), carbon fabric (FC), and carbon pultruded laminate (LC) have been used as FRP. Experimental results and AE behavior are discussed based on an elastoplastic finite-element numerical model. The model demonstrates a strong strain concentration at the end of poplar planks, which causes high AE activity in these areas and acts as a precursor of the delamination of the poplar plank. Based on the experimental results, some AE criteria for predicting the onset of the delamination are tentatively proposed.

## 1. Introduction

The retrofitting and repairing of timber structures is a topic of growing interest, mainly in the case of structures with historical interest, or in those cases where replacing existing structural elements by new ones is extremely expensive and time consuming. Many of the existing wooden structures are made of pine, being the Pinus Sylvester widely used in many areas of the Iberian Peninsula. For several decades, many works have demonstrated the enormous advantages of FRP (fiber reinforced polymer) composite materials for retrofitting and repairing of structural elements (mainly beams) of concrete or timber, whether embedded in the structural element by means of artificially grooves (near-surface mounted technique), or externally applied (usually on the tension face but also on lateral sides of the element for shear reinforcement) [1,2,3,4,5,6,7,8,9,10,11,12,13,14,15,16].

In a previous work [17], some of the authors compared the mechanical behaviour of three FRP retrofitting layouts using basalt fabric, carbon fabric, and carbon laminate, carried out on pine beams and then covered by poplar planks, also used as an additional reinforcement. Retrofitting using only a poplar plank was used as a control layout. By means of an experimental campaign, it was demonstrated that: (1) The MOR (modulus of resistance) achieved by the four layouts is quite similar, around 30 MPa, due to the premature delamination at the poplar plank-FRP interface; (2) the improvement of the stiffness in terms of MOE (modulus of elasticity) is similar using FRP and poplar planks than using only poplar planks. An average increase of 18% of the MOE with respect to the pine beam was achieved; (3) a theoretical analysis based in the parallel axis theorem demonstrated that this improvement is basically provided by the poplar plank, which acts as reinforcement due to its greater stiffness with respect to the pine beam.

Traditionally, the retrofitting of structures with FRP has not been based on numerical or analytical analyses, but mainly on an empirical basis and recommendations from FRP manufacturers. Thus, it is necessary to have monitoring methods both in the medium and long term, in order to obtain information on the behavior of the structure and reinforcements over time, in the context of what is traditionally known as structural health monitoring (SHM). One of the well-known SHM methodologies is the acoustic emission method (AE).

Acoustic emission is the elastic wave released by a material or structure when changes occur in its deformation field. Traditional sources of acoustic emission are the formation and growth of cracks and delamination between materials or components. The acoustic emission waves propagate within the material and when it reaches its surface they can be converted into acoustic signals (acoustic emission signals) using appropriate piezoelectric sensors. Its detection and analysis allow to evaluate and predict the failure. Acoustic emission is a method that fits uniquely to the concept of SHM and the real-time and continuous monitoring of a structure. Some applications of the AE method for the study of wood behavior can be found in [18,19,20,21].

In this paper, a bath of four beams of our previous work [17] (which includes the four retrofitting layouts) was monitored with multi-resonant AE sensors during the bending test, in order to evaluate the potential of the AE technique for predicting the poplar plank delamination and damage monitoring in real applications. Moreover, experimental results and acoustic emission behavior are discussed based on an elastoplastic finite-element numerical model. The numerical simulation requires a mechanical characterization of each material carried out in previous works [17] and provides results that cannot be captured by the experiments [4,18,19,20,21,22,23]. The FEM-model results demonstrate a strong strain concentration at the end of poplar planks, which is the cause of the high acoustic emission activity in these areas and the precursor of the delamination of the poplar plank. Based on the experimental results, some criteria for predicting the beginning of the delamination of the poplar plank based on the AE activity (localized events rate) and the AE intensity (signal strength) are proposed. The effectiveness of these criteria will be analyzed and validated in future works.

## 2. Materials and Methods

### 2.1. Samples

Four Pinus Sylvester wooden beams with a cross section of 90 × 60 mm^2^ and a length of 1240 mm were prepared. The dynamic modulus of elasticity (MOEdin, p) was obtained by means of longitudinal vibration tests, by using a Fakopp SD-02 (Fakopp Entreprise Bt., Agfalva, Hungary) (see Table 1). The pine beams were retrofitted with poplar planks as explained in [17]. Static modulus of elasticity (MOEst, po) of the poplar wood was calculated between 20% and 50% of the MOR, as the slope of the stress-strain plot (Table 1). Tests were carried out at 12% moisture content. Three of them were also retrofitted with a layer of unidirectional FRP placed between the pine element and the poplar plank: (1) Basalt fabric (FB); (2) carbon fabric (FC); (3) and carbon pultruded laminate (LC). Table 2 shows the thickness and the MOE along the fiber of the FRP. More details can be seen in a previous paper by the authors [17]. Samples SP, SFB, SFC, and SLC correspond with RB_P_2, RB_P_FB_2, RB_P_FC_2, and RB_P_LC_3 of that previous paper, respectively.

### 2.2. Experiment: Four-Points Bending Test Monitored with Acoustic Emission

**Figure 1:**
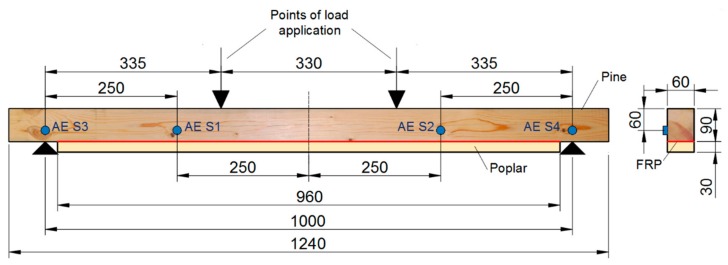
Experimental setup. Distances in mm.

**Figure 2:**
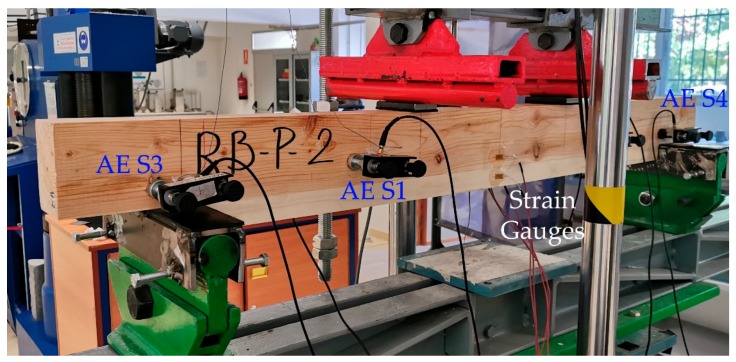
Image of the specimen SP during the bending test.

All the four samples were subjected to a four-point bending test, as shown in Figure 1 and described in [17]. The strains at the mid cross-section were measured by gluing six strain gauges as explained in [17]. In order to obtain the MOEst (static modulus of elasticity), the stress-strain slope, with the strain gauge placed on the tension face of the poplar plank, was calculated between 20% and 50% of the maximum stress. Four multiresonant VS45-H piezoelectric sensors (AE sensors, Vallen Systeme, Icking, Germany) were mounted on each specimen, as indicated in Figure 1 [18]. They were acoustically coupled to the specimens by means of silicone grease, and using magnetic holders hooked to flat washers, which were in turn previously glued on the wood surface. The acquisition threshold was 25 dB and the gain 34 dB. The AE signals were acquired with five Msamples/s, a 50 kHz high pass filter, and a 500 low pass filter. Moreover, in order to avoid friction noise, thin Teflon pieces were placed between the wood specimen and the supports. Figure 2 depicts a particular specimen at the testing machine. For each recorded signal, the peak amplitude in dB (A) and the signal strength (SS) were obtained. Moreover, using the hits received for each sensor, a linear location of the AE events was carried out, and 300 cm/ms was used as propagation speed of the specimen along the longitudinal direction (grain direction).

### 2.3. FEM Simulations

The elastoplastic behavior of the four specimens was analysed by the numerical technique of the finite element method. The geometry of the experimental setup shown in Figure 1 was modeled in AutoCAD 2012 (Autodesk^®^, San Rafael, CA, USA) [24] CAD software and imported into the open source finite element code Salome Meca^®^ (Code Aster) [25] using the IGES format which allows an easy exchange of information between codes, finally results were drawn with MATLAB^®^ (2013a, Mathworks, Natick, MA, USA) [26]. The constitutive parameters (MOE, Poisson’s ratio, elastic limit, and MOR) of pine timber, poplar planks, and FRP were taken from the experiments [17] and shown in Table 3. The points of load shown in Figure 1 were applied as distributed loads in the transverse direction, and the beams were simply supported. Domain was discretized into a finite element mesh composed by 75,000 eight-node hexaedral elements and 91,364 nodes. The average element size was 5 mm, except for the FRP layer. Table 2 shows that the thickness ranges from 0.14 to 1.4 mm. Distributed transverse loads and boundary conditions were transferred into the finite element mesh. Notice that domains of pine beam, FRP layer, and poplar planks are tied by the finite element mesh. The elastoplastic nonlinear analysis was carried out using the linear elastic perfectly plastic model from Salome Meca^®^ (Code Aster, 2018, Électricité de France, Paris, France) [25], where the nonlinear equations were solved with a direct full newton solver. See reference [27] for detailed explanation. Loads are applied incrementally in each step with an increase of 0.001 of the total load. The analysis provides displacements, stresses, strains, and yielding of nodes and elements of the mesh at every step. The set of nodes and elements in the middle of the top and bottom faces and in the position of the gauges described in the previous section are used to compare the finite element results with those provided by the experiments.

## 3. Numerical Simulation Results

**Figure 3:**
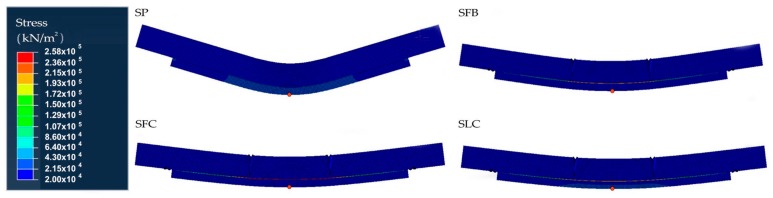
Numerical stress distribution for each specimen.

**Figure 4:**
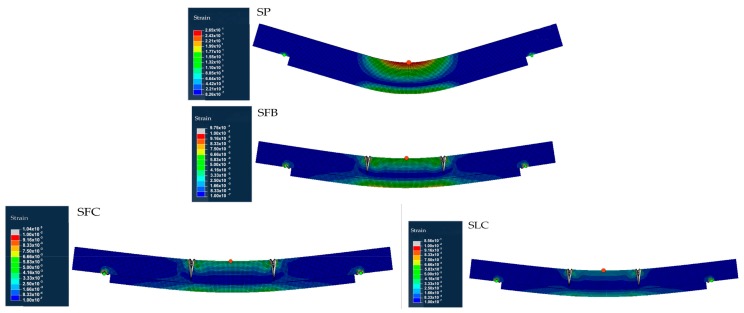
Numerical strain distribution for each specimen.

Figure 3 and Figure 4 show the equivalent stress (Von Mises) and equivalent strain distribution numerically obtained for each particular specimen. Using the normal stress and strains distributions, not shown in this paper for simplicity, we can determine the tension and compression areas. As it can be seen in Figure 3, for the SP beam the maximum tensile stress value is reached at the poplar plank, 25 MPa (see the red point in Figure 3). However, for the SFB, SFC, and SLC specimens, the stress is a 28.8%, 40.1%, and 40.2%, respectively, lower compared to the SP beam (see the red point in Figure 3). This fact is mainly caused by the absorption of stresses carried by the FRP element. The strain field is consistent with that expected, being the central area of maximum compressions and tensile stresses the MOEst demanded. For the SP specimen, there is a concentrate strain area with higher values at the upper part of the mid-span of the specimen. Table 4 summarizes the numerical MOE (MOE_num_) obtained for the four beams, as well as the maximum strains obtained for the upper and bottom face (maximum compression and tensile strains, respectively). In this area, the maximum strain value was of 0.12%, highly different to the 0.08%, 0.08%, and 0.07% reached by the SFB, SFC, and SLC specimens, respectively (see the red point in Figure 4). These lower values of strain for the specimens retrofitted with FRP elements, are correlated with the softer distribution of the strain field along the length of the beam. In addition, for all the beams there is a high strain concentration at the support areas (see green points in Figure 4), being critical points to produce a premature delamination. Regarding the different types of FRP, the maximum compression strain for the SLC specimen was 89.9% and 86.1% lower compared to the SFB and SFC beams, respectively, which is highly correlated with the different stiffness of the FRP materials (see the red point in Figure 4).

**Figure 5:**
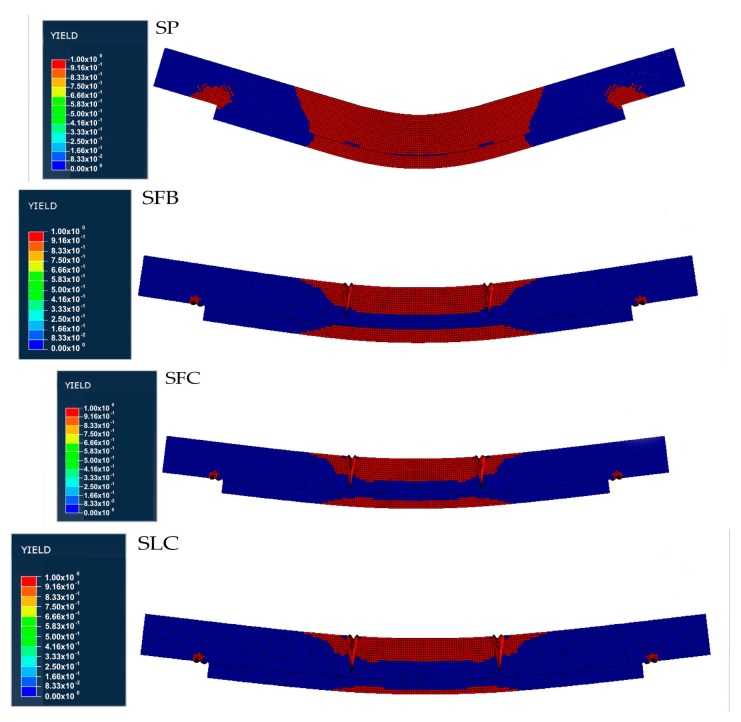
Numerical plasticization pattern for each specimen.

Figure 5 shows the plasticization pattern, demonstrating the previous stress/strain differences between the specimens retrofitted with FRP elements and the SP specimen, as well as the stiffness variations of the different FRP materials. In particular, it can be noticed that the SP specimen reaches the highest plasticization level between the four specimens, with all the mid-part of the specimen plasticized. On the other hand, the SLC specimen is the least plasticized due to the higher stiffness of the CFRP laminate. SFB and SFC ones show a similar behavior.

## 4. Experimental Results

**Figure 6:**
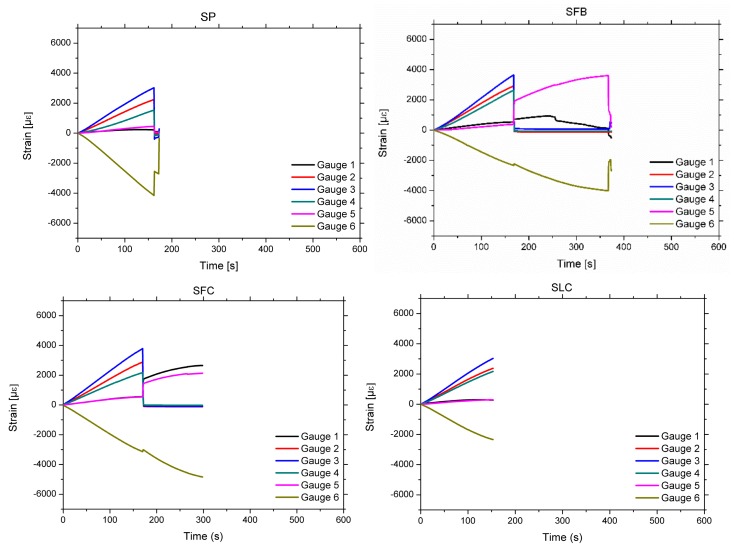
Experimental strain-time plots for all the specimens. See [17] for the position of the strain gauges.

**Figure 7:**
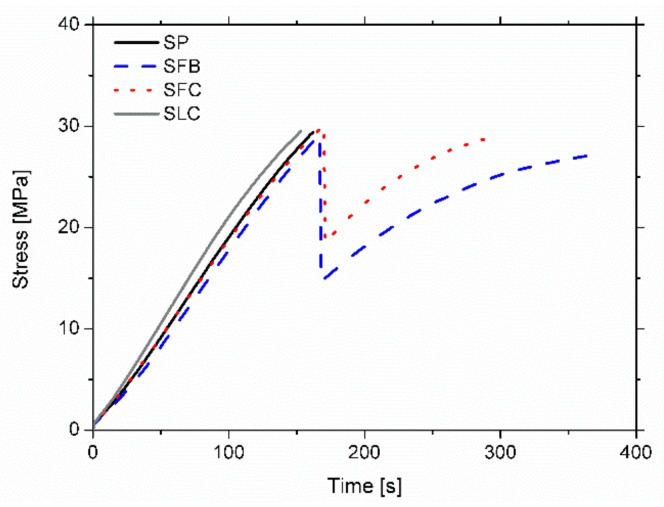
Experimental stress-time plot of tensile strain (gauge 3) for all the specimens.

Figure 6 shows the strain-time plots, the strain being measured by the six gauges for all the four specimens. As expected, the maximum strains are obtained at the upper and bottom external faces (gauge 3 for tensile and gauge 6 for compression strains). Similarly, the strain at points 2 and 4, and 1 and 5, are similar to each other. The strain at the poplar plank (2 and 4) are greater than that at the adjacent points at the pine beam (1 and 5), due to the latter being closer to the neutral fiber. Similarly, Figure 7 shows the stress-time plots for all the specimens, while Table 5 shows the experimental MOEst, exp, and MOR.

The difference between numerical and experimental MOE for the SP beam was of 11.8%, while for the SFB, SFC, and SLC was of −21.3%, −19.9%, and −15.9%, respectively. The assumption that wood is a homogeneous material in the numerical simulations is the main reason of these variations (mainly negatives), since a real timber beam has defects as knots and fiber deviations which can decrease considerably the mechanical properties from an ideal isotropic solid.

**Figure 8:**
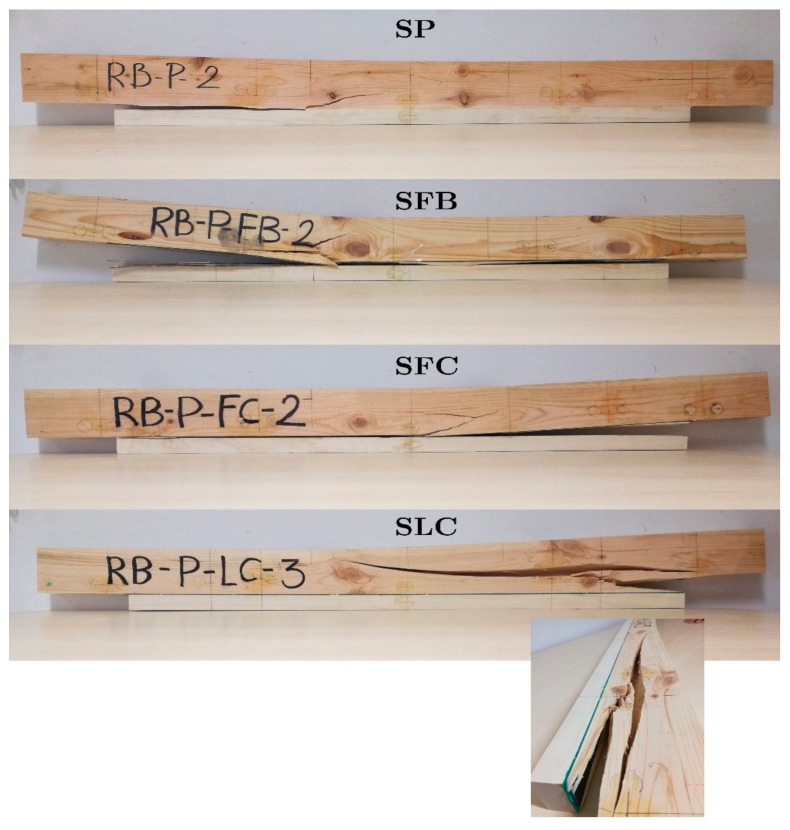
Fracture pattern of the specimens.

For the case of the SP specimen, it can be observed that the strain increases permanently until the end of the test. In this case, the final failure was caused by a strong pine-poplar delamination starting on the left side of the poplar plank, followed by a mixed brittle tensile/shear fracture of the pine located around the mid-span (tension zone of the pine). However, for the case of specimens retrofitted with the fabric FRP (SFB and SFC), the strain increases permanently up to a particular intermediate load point at which a complete delamination between the FRP fabric and the poplar plank occurs. Moreover, at this point there is a sudden drop of the load and the strain, particularly in the tensile area with the strain at the poplar plank becoming very low because it gets detached. After this drop, a gradual new rise of the load occurs until the final brittle fracture of the pine beam (tensile mode for the SFB specimen and mixed tensile/shear mode for the SFC one), is located at the mid-span of the sample. This ductile behavior of the fracture process is very important in terms of safety. Finally, for the sample with LC retrofitting, the strain increases permanently until the end of the test. Final failure occurred by a very slight pine-FRP delamination at the right side of the poplar plank, followed by a strong brittle shear fracture of the pine, which extended along 2/3 of the length of the specimen. Table 5 summarizes the failure modes of the specimens and Figure 8 shows some pictures of the fracture pattern for each particular sample.

## 5. Acoustic Emission Results

**Figure 9:**
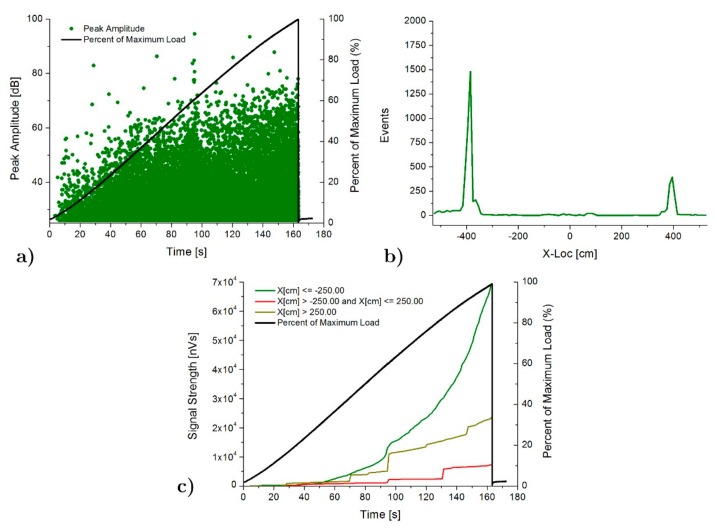
AE results for SP specimen: (**a**) Peak amplitude vs load history over the time, (**b**) Linear location over the specimen and (**c**) Signal strength vs load history over the time.

Figure 9 shows the AE results obtained for the SP specimen. Peak amplitude in dB (Figure 9a) shows a gradual increasing as the load increases until the end of the test, demonstrating a progressive deterioration of the specimen. Second plot, Figure 9b, shows the location results of the AE events as a function of position along the specimen. A clear concentration of events is observed at both ends of the poplar planks, which is clearly due to the strain concentration in these areas, demonstrated by the numerical analysis (Figure 4). Higher concentration is observed on the left side, which is in good agreement with placement of the beginning of pine-poplar delamination occurred at this side, experimentally observed (Figure 8). In order to compare the zonal distribution of the AE intensity, Figure 9c shows the AE signal strength located on the left side (x < −250 mm), right side (x > 250 mm), and central area (−250 < x < 250 mm) of the specimen. Few AE is located in the central area in this case, which appeared mainly at the end of the test.

**Figure 10:**
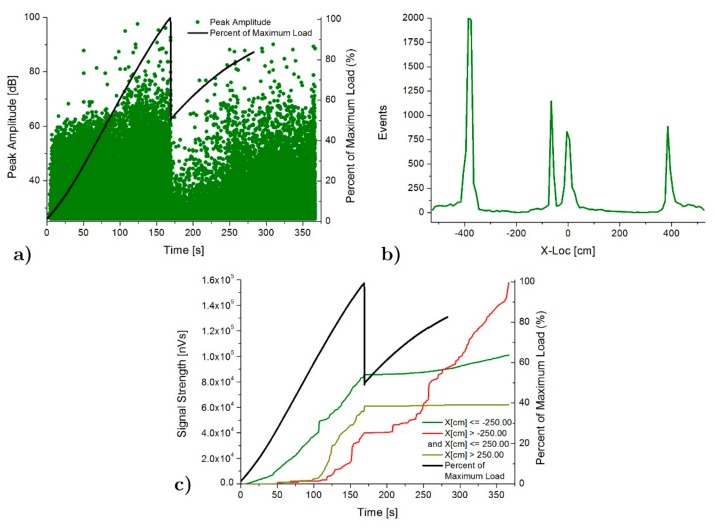
AE results for SFB specimen: (**a**) Peak amplitude vs load history over the time, (**b**) Linear location over the specimen and (**c**) Signal strength vs load history over the time.

**Figure 11:**
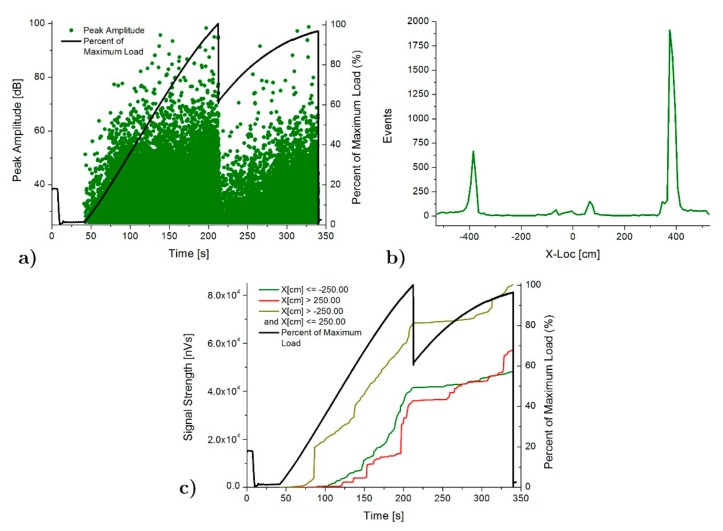
AE results for SFC specimen: (**a**) Peak amplitude vs load history over the time, (**b**) Linear location over the specimen and (**c**) Signal strength vs load history over the time.

Figure 10 and Figure 11 shows the AE results for the case of SFB and SFC specimens, respectively. Peak amplitude in dB shows a gradual increasing as the load increases until the maximum load, just when the FRP-poplar plank delamination happens. At this point, an important drop of the AE intensity also happens (AE peak amplitude decreases from 90 to 40 dB). An increasing of the peak amplitude until the final breakage demonstrates the progressive increase of the deterioration of the pine beam. AE location results show again a clear concentration of events at both ends of the poplar planks due to the strain concentration in these areas, in accordance again with the numerical analysis results. In this case, higher AE activity and intensity is located in the central area of the specimen, compared with that for the SFB one, which is in good agreement with the fact that the delamination between the poplar and the FRP extended along the entire poplar plank. As for the SP specimen, there is a significant growth of the acoustic emission activity in the central area of the specimen after the delamination (starting at t = 210 s for SFB and at 260 s for SFC sample), due to the stress concentration at the maximum bending moment area of the pine, causing the final failure of the specimen.

**Figure 12:**
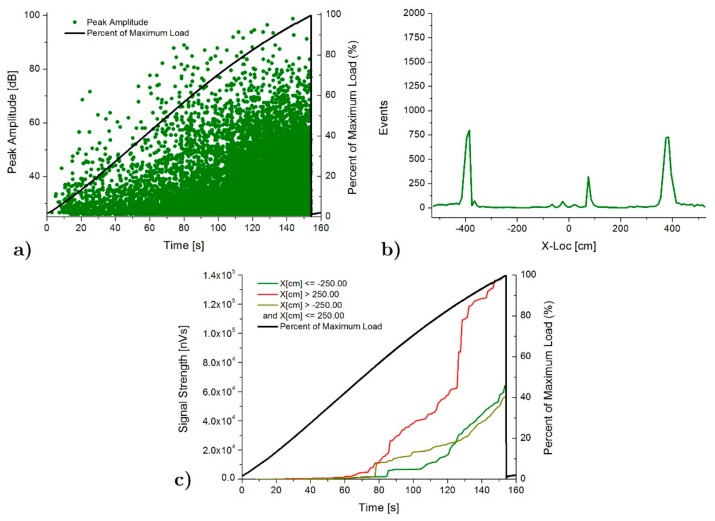
AE results for SLC specimen: (**a**) Peak amplitude vs load history over the time, (**b**) Linear location over the specimen and (**c**) Signal strength vs load history over the time.

Finally, Figure 12 shows the AE results for the specimen retrofitted with pultruded laminates (SLC). In this case, as happens for the SP specimen, the peak amplitude increases until the end of the test, also demonstrating the evolution of the damage. The location of the acoustic emission also coincides with the high strain areas obtained numerically, that is, the end of the poplar planks and the central area of the specimen. However, unlike the rest of the specimens, the acoustic emission activity in the central zone begins earlier than the acoustic emission at the end of the poplar boards. In addition, the intensity and acoustic emission activity at the end of the poplar planks is lower for this specimen than for the specimens SFB and SFC, in line with the fact that the delamination in this case was very small and the failure final was due to shear fracture of the pine beam.

## 6. Discussion and Conclusions

By means of a FEM elastoplastic analysis of pine beams retrofitted with FRP and poplar planks it has been demonstrated that: (i) FRP absorbs most of the stress and provides a softer distribution of the strains field; (ii) a strong strain concentration is observed in poplar tensile and pine compression areas, but also at the end of the poplar planks (near the support areas), demonstrating that these are the most critical areas to initiate a delamination between FRP and poplar plank, as validated experimentally; (iii) FRP provides a reduction of the maximum deformations with respect to the retrofitting layout using only the poplar plank,; (iv) LC layout undergoes the lower maximum deformations than fabric (carbon or basalt) fabric layouts; (v) the specimen without FRP, i.e., only retrofitted with the poplar plank, suffers the largest plasticized area, while the specimen retrofitted with pultruded laminate, SLC, suffers the smallest plasticized area.

Experimentally, it has been verified that the SLC specimen did not show a relevant FRP-poplar plank delamination. In contrast, a strong partial or complete delamination between FRP and poplar plank preceded the final breakage of the pine beam for the rest of the specimens (SP, SFB, or SFC), probably due to the lower deformations and plasticization area, and a poorer adherence. As expected, a higher value of MOE was obtained for the SLC specimen, compared with SP, SFB, and SFC.

Acoustic emission showed a clear increase in terms of both peak amplitude (intensity) and event rate (activity) features from the beginning of the load until the FRP-poplar plank delamination occurrence. Using the SLC as a “reference specimen”, 6104 ue and 800 events/dm could be established as nondelamination reference values for the accumulated signal strength and located-event rate features, respectively. Thus, higher values of these AE features could indicate the appearance of delamination. Using these criteria, the delamination in Table 6 can be built, which is in good agreement with experimental observations of the fracture patterns. These results demonstrate that acoustic emission is a promising technique for the in situ monitoring of wood structural elements retrofitted with wood and/or FRP materials. However, the authors are aware of the limited number of specimens tested in this study. For its standardization, larger test campaigns are required in the future.

## Figures and Tables

**Table 1:** Mechanical properties of pine elements and poplar planks used for the specimens.

Specimen	MOE_din_, p	MOE_st_, po
SP	6465	10,261
SFB	7709	9441
SFC	7186	9441
SLC	8775	10,142

**Table 2:** FRP properties for each particular specimen.

Configuration	Reinforcement Type	FRP Thickness (mm)	Resistant Area per Unit Width (mm^2^/m)	FRP Elastic Modulus (GPa)
SP	Poplar	−	−	−
SFB	FB/Poplar	0.14	142.5	890
SFC	FC/Poplar	0.17	166.6	2300
SLC	LC/Poplar	1.4	−	1650

**Table 3:** FRP properties for each particular specimen.

Reinforcements	Elastic Modulus (MOE) kN/m^2^	Poisson’s Ratio	Yield Stress kN/m^2^	(MOR) kN/m^2^
Poplar	8.8 × 10^6^	0.37	2 × 10^4^	3.8 × 10^4^
Carbon fabric (FC)	2.3 × 10^8^	0.30	3 × 10^6^	4.83 × 10^7^
Basalt fabric (FB)	8.9 × 10^7^	0.30	2 × 10^6^	4.84 × 10^7^
Carbon pultruded laminate (LC)	1.65 × 10^8^	0.30	2.5 × 10^6^	2.6 × 10^7^

**Table 4:** Mechanical properties of each specimen numerically obtained.

Specimen	MOE_num_ (MPa)	Maximum Strain Upper Face (pine) (%)	Maximum Strain Bottom Face (poplar) (%)
SP	8455	0.124	0.255
SFB	9901	0.078	0.189
SFC	9807	0.081	0.159
SLC	11309	0.070	0.159

**Table 5:** Mechanical properties of each specimen experimentally obtained.

Specimen	MOE_st,exp_	MOR	Failure Pattern
SP	9590	29.4	Strong pine-poplar delamination and a mixed brittle tensile/shear fracture of the pine at the end of the test
SFB	8160	29.1	Complete FRP-poplar delamination at 46% and a brittle tensile fracture of the pine at the end of the test
SFC	8180	30.1	Complete FRP-poplar delamination at 58% and a mixed brittle tensile/shear fracture of the pine at the end of the test
SLC	9760	29.5	Very slight poplar-FRP delamination and a brittle shear fracture of the pine at the end of the test

**Table 6:** Delamination criteria based on AE futures: SS (signal strength), located events rate.

Specimen	Left Side		Right Side		Delamination Percentage
	SS (10^4^ ue)	Events Rate (events/dm)	SS (10^4^ ue)	Events Rate (events/dm)	
SP	7	1500	2.3	400	100%
SFB	10	2000	6	900	100%
SFC	5	680	8.5	1900	100%
SLC	6.2	800	5.8	720	10%
